# Aberrant Brain Spontaneous Activity and Synchronization in Type 2 Diabetes Mellitus Subjects Without Mild Cognitive Impairment

**DOI:** 10.3389/fnins.2021.749730

**Published:** 2021-12-16

**Authors:** Yifan Li, Mingrui Li, Yue Feng, Xiaomeng Ma, Xin Tan, Yuna Chen, Chunhong Qin, Haoming Huang, Yi Liang, Shijun Qiu

**Affiliations:** ^1^First Clinical Medical College, Guangzhou University of Chinese Medicine, Guangzhou, China; ^2^Department of Radiology, The First Affiliated Hospital of Guangzhou University of Chinese Medicine, Guangzhou, China

**Keywords:** type 2 diabetes mellitus, resting-state, percent amplitude of fluctuation, functional connectivity, cognitive function

## Abstract

**Objective:** We aimed to explore whether the percent amplitude of fluctuation (PerAF) measurement could provide supplementary information for amplitude of low-frequency fluctuation (ALFF) about spontaneous activity alteration in type 2 diabetes mellitus (T2DM) subjects without mild cognitive impairment (MCI). Then we further evaluated the synchronization through the method of functional connectivity (FC) to better demonstrate brain changes in a more comprehensive manner in T2DM.

**Methods:** Thirty T2DM subjects without MCI and thirty well-matched healthy subjects were recruited in this study. Subjects’ clinical data, neuropsychological test results, and resting-state functional magnetic resonance imaging (rs-fMRI) data were acquired. Voxel-based group difference comparisons between PerAF and ALFF were conducted. Then, seed-based FC between the recognized brain regions based on PerAF and ALFF and the rest of the whole brain was performed.

**Results:** Compared with healthy group, T2DM group had significantly decreased PerAF in the bilateral middle occipital gyrus and the right calcarine, increased ALFF in the right orbital inferior frontal gyrus and decreased ALFF in the right calcarine. Seed-based FC analysis showed that the right middle occipital gyrus of T2DM subjects exhibited significantly decreased FC with the right caudate nucleus and right putamen. According to the partial correlation analyses, hemoglobin A1c (HbA1c) and immediate memory scores on the auditory verbal learning test (AVLT) were negatively correlated in the T2DM group. However, we found that total cholesterol was positively correlated with symbol digit test (SDT) scores.

**Conclusion:** PerAF and ALFF may have different sensitivities in detecting the abnormal spontaneous brain activity in T2DM subjects. We suggest PerAF values may add supplementary information and indicate additional potential neuronal spontaneous activity in T2DM subjects without MCI, which may provide new insights into the neuroimaging mechanisms underlying early diabetes-associated cognitive decline.

## Introduction

Type 2 diabetes mellitus (T2DM) can lead to cognitive impairment, which mainly presents as memory deterioration, slowed information processing speed and executive function (Cui et al., 2014) and can even progress to dementia. Compared to healthy aging people, T2DM subjects are more prone to accelerated cognitive decline (Xiong et al., 2020). Hence, early and accurate diagnosis of T2DM-related brain decline can improve their quality of life.

According to previous studies, many cognitive functions gradually decline before significant mild cognitive impairment (MCI) occurs (Ma et al., 2015; Yang et al., 2018; Li et al., 2020). Our previous studies revealed that white matter microstructure has altered in T2DM subjects without MCI in the bilateral cingulum lobe and thalami, pons, and left temporal pole (Liang et al., 2019). It has been reported that changes in brain structure may contribute to changes in brain function (Cui et al., 2021). Therefore, we studied early neural spontaneous activity before MCI to determine the neural mechanisms of cognitive changes and investigate the imaging biomarkers of early cognitive decline in T2DM subjects.

In the last few years, many studies have proposed that abnormal neural spontaneous activity might be the internal mechanism for cognitive impairment in T2DM subjects based on results obtained using the amplitude of low-frequency fluctuation (ALFF) method (Liu et al., 2021). Compared to healthy controls (HCs), T2DM subjects have lower ALFF in some regions, such as the frontal lobe, inferior parietal lobule, precuneus, posterior cingulate gyrus, and posterior cerebellar lobe (Wang et al., 2011; Xia et al., 2013; Cui et al., 2014). However, none of these studies focused on early changes, and the included subjects were all relatively elderly. In addition, these studies have produced inconsistent results, probably because ALFF is too sensitive to use for subsequent group-level statistical analysis firsthand (Jia et al., 2020). At present, Jia et al. (2020) have proposed the percent amplitude of fluctuation (PerAF) method of assessment, which evaluates the percentage of blood oxygen level-dependent (BOLD) signal fluctuations with high test-retest reliability. From the method point of view, ALFF directly measures the regional spontaneous brain activity of a specific voxel, because it shows direct performance of the spontaneous brain activity in each voxel. The amplitude of any random frequency is proportional to the square of the amplitude of this frequency component of the whole original time series, the square root is calculated at each frequency of the power spectrum and the averaged square root is obtained across 0.01–0.08 Hz at each voxel. This averaged square root is defined as the ALFF. Therefore, the ALFF value is proportional to the mean value of the time series. However, the absolute BOLD signal intensity has arbitrary units, ALFF results will be affected by the scale of BOLD signal. PerAF can avoid that. PerAF, which named percentage amplitude fluctuation, measures the percentage of BOLD fluctuations relative to the mean BOLD signal intensity for each time point and averages across the whole time series. As compared with ALFF, PerAF is a scale-independent method. It’s a similar metric to the percent signal change can be formulated for resting-state functional magnetic resonance imaging (rs-fMRI). In addition, PerAF can prevent confounding factors of voxel-specific fluctuation amplitudes. Therefore, PerAF may be a potential measurement of voxel-level spontaneous BOLD activity that is crucial for investigating cognitive function decline in T2DM subjects. The proposed PerAF may increase sensitivity and reduce bias when evaluating the neural spontaneous activity alterations in T2DM subjects. Seed-based functional connectivity (FC) has been used to assess cerebral activity synchronization in T2DM subjects (Cui et al., 2016; Liu et al., 2019). Seed-based FC selects the voxel or seed region empirically and is used for calculating correlations with the remaining voxels in the brain. This method could directly indicate the regions most connected with the seed region and be used in numerous studies of T2DM-related cognitive mechanisms.

Based on this evidence, neural spontaneous activity and synchronization were assessed with PerAF and FC, and the results may contribute to a better understanding of the mechanisms of early cognitive decline in T2DM subjects. Research in the field of T2DM-related cognitive impairment has increased. However, some research has inconsistent results. For example, many studies have suggested that T2DM subjects have decreased FC in default mode networks (Hoogenboom et al., 2014; Yang et al., 2016). There is also research indicating that the hippocampus, which is prone changes caused by T2DM, is a crucial factor in the decrease of FC in other regions (McCrimmon et al., 2012). Moreover, the discrepant results across studies may reflect different levels of cognitive decline. We took a closer look at this discrepancy measured by PerAF and FC. This study mainly focused on the potential undiscovered changes in T2DM subjects without MCI. Pertinently, these changes are exactly what we need to pay attention to and study because intervention in the earlier brain changes can lead to greater improvements in a patient’s prognosis and indicates the mechanism of early neurologic changes.

## Materials and Methods

### Subjects

We investigated early changes in T2DM subjects without clinically significant cognitive decline or any significant brain diseases. This study was examined and approved by the Medical Research Ethics Committee of Guangzhou University of Chinese Medicine. Written informed consent was provided by all subjects. T2DM subjects were enrolled from the inpatients of the endocrinology department in The First Affiliated Hospital of Guangzhou University of Chinese Medicine from January 1, 2018, to January 30, 2020 (Table 1). All subjects met the following requirements: age between 40 and 60 years, years of education >6, and right-handed. T2DM subjects met the diagnostic criteria released by the American Diabetes Association (ADA) in 2018 and were treated with insulin therapy via insulin pump or insulin injections. Demographically matched HCs were recruited for comparison; the two groups were matched with respect to age, sex, education, and handedness. After asking the subjects about their medical history and going through a standard set of questionnaires, subjects meeting the following criteria were excluded: a diagnosis of other types of diabetes, history of alcohol or substance abuse, significant head trauma, psychiatric or neurological disease that affects central nervous system function, any systemic disease (e.g., tumor and meningitis) and claustrophobia. Because this study only focused on T2DM subjects without MCI, subjects who scored below 26 on the Montreal Cognitive Assessment (MoCA, Beijing edition) were not enrolled (Li et al., 2018). Ten participants were excluded because their MoCA scores did not meet the requirements. Six more subjects were excluded because of image-related problems, such as substantial motion (>2 mm translation or > 2 rotation in any direction) or image artifacts. Finally, a total of 60 participants (30 T2DM subjects and 30 HCs) were included in this study.

**TABLE 1:** Demographics, clinical data, cognitive assessment of T2DM and HCs.

Characteristics	T2DM subjects (n = 30)	Healthy controls (n = 30)	Statistics	*P*-value
**Demographics**				
Age (years)	49.23 ± 5.52	45.77 ± 6.43	*t* = 2.242	0.341
Sex (male/female)*				
Education	12 (6, 16)	9 (6, 16)	*z* = −0.608	0.543
**Clinical data**				
BMI	25.01 ± 2.48	22.66 ± 2.39	*t* = 3.733	0.531
SBP	132.93 ± 15.68	118.37 ± 11.34	*t* = 4.124	0.179
DBP	85.73 ± 10.79	78.9 ± 7.46	*t* = 2.854	0.084
HbA1c (%)	8.70 ± 2.19	N/A	N/A	N/A
FBG (mmol/L)	7.41 ± 1.78	N/A	N/A	N/A
FINS (μIU/ml)	5.94 (0.79, 18.9)	N/A	N/A	N/A
TG (mmol/L)	1.6 (0.64, 7.61)	N/A	N/A	N/A
TC (mmol/L)	4.51 ± 1.04	N/A	N/A	N/A
LDL (mmol/L)	2.96 (1.47, 5.81)	N/A	N/A	N/A
**Cognitive assessment**				
MoCA score	26.5 (26, 29)	28.5 (26, 30)	*z* = −4.319	0.000*
AVLT(immediate)	20.47 ± 4.82	21.63 ± 5.51	*t* = −0.873	0.399
AVLT(5 min)	7.67 ± 1.88	8 (4,14)	*z* = −1.406	0.160
AVLT(20 min)	7.77 ± 2.40	8.53 ± 2.28	*t* = −1.266	0.622
AVLT(recall)	12 (6, 12)	12 (5, 13)	*z* = −1.182	0.237
Grooved pegboard (R)	80.96 ± 17.41	72.20 ± 10.91	*t* = 2.333	0.108
Grooved pegboard (L)	85.26 ± 13.87	79.62 ± 12.86	*t* = 1.633	0.692
TMT-A	53.5 (21, 156)	51.41 ± 15.61	*z* = −1.346	0.178
TMT-B	43.5 (19, 118)	40 (23.4, 89)	*z* = −0.932	0.351
DST(forward)	8.07 ± 1.55	8 (6, 11)	*z* = −0.841	0.401
DST(inverse)	4 (2, 10)	4 (2, 10)	*z* = −0.628	0.530
CDT	3 (2, 3)	3 (2, 3)	*z* = −1.390	0.165
SDT	43.23 ± 9.71	52.47 ± 14.73	*t* = −2.866	0.020*

*Values distributed normally or non-normally are presented as the mean ± SD or median (minimum and maximum).*

BMI, body mass index; SBP, systolic blood pressure; DBP, diastolic blood pressure; FBG, fasting blood glucose; FINS, fasting insulin; TG, triglyceride; TC, total cholesterol; LDL, low-density lipoprotein; MoCA, Montreal Cognitive Assessment; AVLT, auditory verbal learning test; TMT, trial-making test; GPT, grooved pegboard test; SDT, symbol digit test; CDT, clock drawing test; DST, digital span test. N/A, not applicable. *P < 0.05.

### Biometric Measurements

Detailed clinical data were collected, including age, sex, blood pressure (BP), body mass index (BMI), years of education, and medication. Participants’ BP was checked at three different times in 1 day, and the results were averaged. Fasting blood glucose (FBG), hemoglobin A1c (HbA1c), low-density lipoprotein (LDL), total cholesterol (TC), and triglycerides were measured in the morning after overnight fasting.

### Cognitive Assessment

A range of neuropsychological assessments that covered the overall T2DM-related cognitive field were conducted, including the MoCA (Kawada, 2019), auditory verbal learning test (AVLT) (Zhao et al., 2015), trail making test (TMT; including parts A and B) (Camilleri et al., 2015), grooved pegboard test (GPT) (Tolle et al., 2019), symbol digit test (SDT) (Silva et al., 2019), clock drawing test (CDT) (Zhou et al., 2010), and digital span test (DST, including forward and backward) (Diamond, 2013). These scales are widely used to assess cognitive function in the study of cognitive mechanisms related to T2DM (Yang et al., 2018; Li et al., 2020). This battery of assessments took 50 min to complete.

### Resting-State Functional Magnetic Resonance Imaging Data Acquisition

Resting-state functional magnetic resonance imaging data were acquired at The First Affiliated Hospital of Guangzhou University of Chinese Medicine on a 3-T GE SIGNA MRI scanner with an 8-channel head coil. Subjects lay supine on the scanning bed and had to stay awake and keep their eyes closed during the scanning process. Participants also wore earplugs to block out noise, and a foam pad was used to keep their heads stationary during the scanning process. Conventional sequences were performed first to screen brain lesions, including axial T1-weighted and fluid-attenuated inversion recovery (FLAIR) images. Two radiologists who had more than 5 years of working experience and who were blinded to the subject information inspected the conventional images to rule out intracranial lesions. None of the participants were excluded based on the abovementioned exclusion criteria. Then, the experimental sequences were subjected to data processing. The parameters of the sequences were consistent with those of our previous study (Tan et al., 2019).

### Data Processing

Resting-state functional magnetic resonance imaging data were preprocessed using the MATLAB R2014a platform and analysis toolkit RESTplus V1.21^1^ (Jia et al., 2020). The preprocessing procedure included removing the first 10 volumes, slice timing, realignment, normalization to Montreal Neurological Institute (MNI) space and re-sampled at a resolution of 3 mm× 3 mm× 3 mm, smoothing (full-width Gaussian kernel = 6 × 6 × 6 mm), detrending and covariance regression. Linear regression was used to remove the covariates of global mean signal, white matter, head-motion, and cerebrospinal fluid signal. All participants’ images were within the defined motion thresholds (i.e., less than 2 mm in translation or more than 2 in rotation in any direction).

The ALFF, PerAF, and seed-based FC methods were conducted successively based on the preprocessed images using RESTplus. ALFF was performed after filtering (0.01–0.08 Hz). The brain regions with altered ALFF and PerAF measurements determined by comparison between the two groups were regarded as seed regions. Peak MNI coordinates of the seed regions were regarded as the center of the region of interest (ROI) with a 6-mm radius. Then, the average values across the time series of seed regions were extracted and correlated with each voxel of the rest of the brain. Fisher’s r-to-z transformation was conducted to increase the normality of the data.

### Statistical Analysis

#### Demographic, Biochemical, and Cognitive Characteristics Analysis

SPSS software (version 22.0; Chicago, IL, United States) was used to compare the differences in demographics, clinical variables, and cognitive performance between the HC group and T2DM group. The Kolmogorov–Smirnov test was conducted to evaluate whether the data, which comprised the demographic data and clinical characteristics of all subjects, met the criteria for a Gaussian distribution. Independent two-sample *t*-test was used for normally distributed data with homogeneity of variance, and Mann–Whitney non-parametric test was used for the remaining data. Chi-square tests were performed for proportions. *P* < 0.05 was considered to be statistically significant.

#### Intergroup Comparison of Resting-State Functional Magnetic Resonance Imaging Indicator

Independent two-sample *t*-test was performed using RESTplus data analysis kit V1.21 software (within a 61 × 73 × 61 mm brain mask) to measure intergroup comparisons of PerAF and ALFF in brain alterations. Gaussian random field (GRF) correction with voxelwise *p* < 0.01 and cluster-level *p* < 0.05 was set. Intergroup comparisons of FC were also GRF-corrected with the same correction parameters.

### Correlation Analysis

Partial correlation analysis was performed to explore the association between cognitive performance and diabetes-related parameters after controlling for sex, age, and education level. Mean ALFF, PerAF, and FC signal values for each brain region with significant differences were extracted. Pearson’s correlation analysis evaluated associations among the mean *z*-values, neuropsychological test scores and clinical variables. Significant thresholds were set to *p* < 0.05.

## Results

### Demographic, Clinical and Neuropsychological Results

The demographic, clinical and neuropsychological information of all subjects was presented in Table 1. There was no significant difference in age, sex, or education levels between the two groups. The T2DM subjects showed significantly worse performance on the MoCA and SDT tests than HCs (*p* < 0.05).

### Intergroup Comparison in Percent Amplitude of Fluctuation and Amplitude of Low-Frequency Fluctuation

**FIGURE 1:**
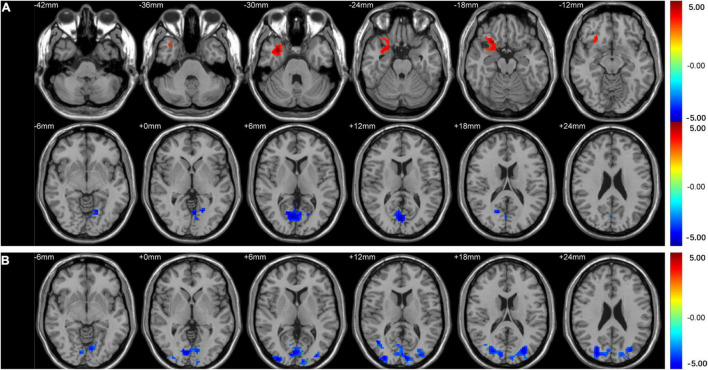
Clusters of between-group differences in ALFF and PerAF adjusted for age, sex, and education level (GRF corrected, voxel-wise *p* < 0.01 and cluster-level *p* < 0.05). Significantly higher regional ALFF values were found in the right orbital inferior frontal gyrus, decreased ALFF values were found in the right calcarine **(A)**, and significantly decreased PerAF values were found in the bilateral middle occipital gyrus and right calcarine **(B)** in T2DM subjects compared to HCs.

Intergroup comparison showed that compared with HCs, T2DM subjects had significantly increased ALFF in the right orbital inferior frontal gyrus, decreased ALFF in the right calcarine (Table 2 and Figure 1A), and decreased PerAF in the bilateral middle occipital gyrus and the right calcarine (Table 2 and Figure 1B).

**TABLE 2:** Brain regions with decreased PerAF in T2DM subjects compared with HCs.

Indicator	Cluster	Brain regions	MNI coordinates			Voxels	*t*-value
			x	y	z		
ALFF	1	Right orbital inferior frontal gyrus	33	6	−30	205	4.3409
	2	Right calcarine	3	−78	9	248	−3.7532
PerAF	1	Bilateral middle occipital gyrus right calcarine	27	−84	21	659	−4.107

*PerAF, percent amplitude of fluctuation; MNI, Montreal Neurological Institute; X, Y, and Z, coordinates of primary peak locations in MNI space.*

### Intergroup Comparison in Functional Connectivity

**FIGURE 2:**
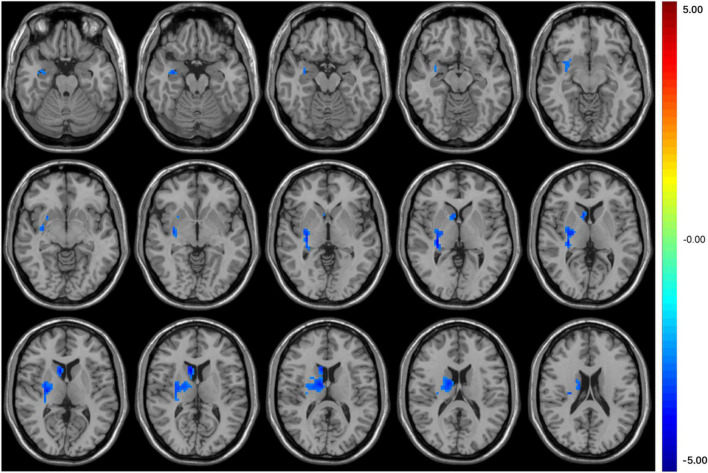
The colored brain regions represent significantly decreased FC between the middle occipital gyrus and right basal ganglia (caudate nucleus and putamen) in T2DM patients compared with HCs. The color bar indicates the *t*-value from two-sample *t*-tests.

Seed-based FC analysis showed that compared with HCs, T2DM subjects exhibited significantly decreased FC between the right middle occipital gyrus and the right basal ganglia, which contains the right caudate nucleus and right putamen (Table 3 and Figure 2). However, there was no correlation between the right orbital inferior frontal gyrus/the right calcarine and the rest of the brain regions in T2DM subjects.

**TABLE 3:** Brain regions with increased FC in T2DM subjects compared with HCs.

Cluster	Brain regions	MNI coordinates			Voxels	*t*-value
		x	y	z		
1	Caudate_R Putamen_R	9	12	12	292	−3.6663

*FC, functional connectivity; GRF correction (P < 0.01, voxel P < 0.01, cluster P < 0.05). X, Y, Z: coordinates of primary peak locations in MNI space.*

### Partial Correlation Analysis

**FIGURE 3:**
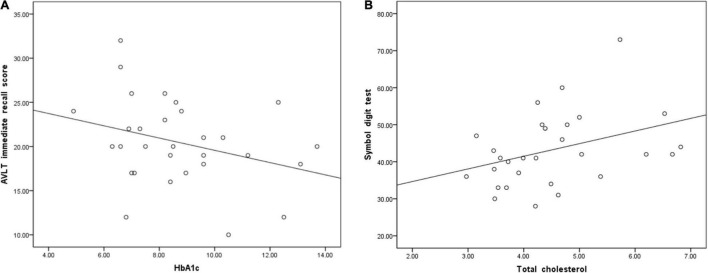
**(A)** Glycosylated hemoglobin A1c (HbA1c) was negatively correlated with immediate memory scores on the auditory verbal learning test (AVLT) (*P* = 0.045, *r* = –0.316). **(B)** Total cholesterol was positively correlated with the symbol digit test (SDT) score (*P* = 0.047, *r* = 0.366).

According to the partial correlation analyses, HbA1c and AVLT immediate memory scores were negatively correlated in the T2DM group (*P* = 0.045, *r* = −0.316, Figure 3A). However, we found that TC was positively correlated with SDT in the T2DM group (*P* = 0.047, *r* = 0.366, Figure 3B).

### Pearson Correlation Analysis

In the T2DM group, correlations were not found between the values of these image indicators and cognitive assessment scores.

## Discussion

As far as we are aware, this is the first study that comprehensively compared PerAF and ALFF values in imaging-based clinical research and the first study to use PerAF to explore the altered brain regions associated with early neural abnormalities in T2DM subjects without MCI. The results manifested that ALFF and PerAF had different sensitivities to T2DM-related abnormal neural activity in the between-group comparison, and both indicators provided complementary information in detecting of neural abnormality changes in T2DM subjects. In T2DM subjects, the right calcarine was an overlapping brain area revealed by both ALFF and PerAF. However, there was a broader scope with decreased PerAF values in regions, including the bilateral middle occipital gyrus. We found increased ALFF values in T2DM subjects in the right orbital inferior frontal gyrus, while PerAF values had no such changes. We believe that the different results obtained by the two methods are due to their different definitions and calculation methods, as previously mentioned. In contrast to ALFF, PerAF is a scale-independent approach that can be used for statistical analysis at the group level. PerAF refrains from the confounders created by the voxel-specific fluctuation amplitude. In general, PerAF is a stable and new approach that can be used to explore neural spontaneous activity (Zhao et al., 2018).

### Alteration in Occipital Regions in Type 2 Diabetes Mellitus Subjects

The decreased PerAF values may indicate that BOLD signal fluctuations were reduced. In the current study, PerAF values of the bilateral middle occipital gyrus and right calcarine were significantly decreased, which implies decreased neural activity.

Previous studies revealed that decreased spontaneous neural activity in the occipital lobe was relevant to impaired visual performance (Cui et al., 2014). Also, T2DM subjects are prone to retinal damage (Yao et al., 2021). The occipital cortex is generally deemed to dominate visual function, especially the primary visual cortex (Wandell, 1999). The altered spontaneous activity in the occipital cortex may suggest visual information processing dysfunction in T2DM subjects. It has been reported that there is aberrant spontaneous neural activity in the occipital cortex in T2DM subjects, which is consistent with our research (Xia et al., 2013; Cui et al., 2014).

In the current study, T2DM subjects did not have alterations in visual function, implying that decreased spontaneous neural activity may be an early sign that occurs before the appearance of clinically measurable symptoms. It would be interesting to follow these subjects over time to explore the clinical significance of these findings.

Strong activation of the visual cortex is associated with motor execution (Lebon et al., 2018). This relationship between spontaneous nerve activity and cognitive function supports our hypothesis that spontaneous neural activity has a significant impact on cognitive function in T2DM subjects.

### Functional Connectivity Alteration in Type 2 Diabetes Mellitus Subjects

It is necessary to explore how the various brain areas work together to better investigate encephalic function (Liu et al., 2018). Decreased FC suggests that temporal consistency between the two brain regions has been reduced. In the present study, compared with the HC group, the T2DM group exhibited significantly decreased FC between the right middle occipital gyrus, which was measured by PerAF, and the right basal ganglia (caudate nucleus and putamen). However, there was no correlation between right orbital inferior frontal gyrus/right calcarine which was measured by ALFF and the rest of the brain regions.

The caudate nucleus is involved not only in complicated emotional regulation but also in executive function (Yang et al., 2015; Huang et al., 2016). Recent evidence has suggested that altered FC of the middle occipital gyrus and caudate nucleus may represent deficiencies in visual information processing and executive function in T2DM subjects (Liu et al., 2020). The putamen is involved in learning and motor control, including language and cognitive functions (Ghandili and Munakomi, 2021). Also, the caudate nucleus and putamen are important parts of the basal ganglia and are responsible for motor function regulation (Andres and Darbin, 2018; Hu et al., 2019). More Pertinently, the basal ganglia structure has been reported to dominate motor function. However, research now shows that the basal ganglia also deal with more complex goal-directed behavior, especially cognitive function (Haber, 2016). We infer that changes in these brain regions contribute to the early decline in cognitive function observed.

### Assessment of Cognitive Function in Type 2 Diabetes Mellitus Subjects

In the neuropsychological results analysis, the T2DM subjects showed significantly worse performance on the MoCA and SDT. The MoCA mainly evaluates memory state, language ability, visual condition, and abstract thinking ability. The SDT measures learning ability, recognition speed and flexibility. The reduced MoCA and SDT scores reflect that decreased cognitive function manifested in the incipient stage of T2DM.

Many studies have found a negative correlation between HbA1c levels and cognitive functions (Abbatecola et al., 2010; Reynolds et al., 2010), including memory (Ravona-Springer et al., 2014), executive function (Munshi et al., 2006), attention and information processing speed (Cukierman-Yaffe et al., 2009). In particular, a systematic review provided evidence that high concentrations of HbA1c were negatively associated with cognitive function in T2DM subjects without dementia (Geijselaers et al., 2015). In this study, HbA1c levels were negatively correlated with AVLT immediate recall scores, indicating that increased HbA1c levels may be a risk factor for early cognitive decline in T2DM subjects.

However, TC was positively correlated with SDT scores. Most of the T2DM subjects’ TC levels were below 7 mmol/L, which may suggest that cognitive function improve as cholesterol levels rise within the normal range. There was no correlation between the remaining clinical variables and cognitive scores. The reason for this lack of correlation may be that since all the subjects scored higher than 26 on the MoCA test, their overall cognitive level was considered in the normal range, and the correlation was not strong enough to be detected. In addition, these factors may not major causes that cause early cognitive decline, at least in the present research.

## Limitation

Several limitations in this study should be indicated. First, the sample size was small. Second, it was a cross-sectional study, so long-term cerebral outcomes could not be evaluated (Geijselaers et al., 2015). Finally, the exact biological mechanism of PerAF remains unclear, so a large number of studies in the future are needed to verify the reliability of this indicator. Although there are some limitations, the positive results are promising and valuable to future research.

## Conclusion

Our study indicated that PerAF and ALFF had different sensitivities in detecting the abnormalities in spontaneous neural activity in T2DM subjects. Combination of PerAF and ALFF could provide complementary information and better elaborate the potential changes in brain function due to T2DM. In summary, the combination of the PerAF and FC analyses in this study revealed that spontaneous activity and synchronization decreased in the right occipital lobe and right basal ganglia before obvious cognitive impairment occurred in T2DM subjects. The findings may provide new insights that improve the present understandings of the mechanisms of T2DM-related cognitive dysfunction from a neuroimaging perspective.

## Data Availability Statement

The raw data supporting the conclusions of this article will be made available by the authors, without undue reservation. Requests to access these datasets should be directed to YLi, miyo382905442@163.com.

## Ethics Statement

This study was examined and approved by the Medical Research Ethics Committee of Guangzhou University of Chinese Medicine. The patients/participants provided their written informed consent to participate in this study.

## Author Contributions

YLi designed the whole experiment and wrote the manuscript. ML, YF, and XM were responsible for collecting imaging data. XT, YC, CQ, and HH contributed to the statistical analysis. YLia and ML revised the manuscript. SQ is the director of this study and provided guidance throughout. All authors contributed to the final version of the manuscript and approved the final manuscript.

## Conflict of Interest

The authors declare that the research was conducted in the absence of any commercial or financial relationships that could be construed as a potential conflict of interest.

## Publisher’s Note

All claims expressed in this article are solely those of the authors and do not necessarily represent those of their affiliated organizations, or those of the publisher, the editors and the reviewers. Any product that may be evaluated in this article, or claim that may be made by its manufacturer, is not guaranteed or endorsed by the publisher.

## Footnotes

1
http://www.restfmri.net
